# A case for the study of native extracellular vesicles

**DOI:** 10.3389/fonc.2024.1430971

**Published:** 2024-07-12

**Authors:** Dhanya Nambiar, Quynh-Thu Le, Ferdinando Pucci

**Affiliations:** ^1^ Department of Radiation Oncology, Stanford University, Stanford, CA, United States; ^2^ Otolaryngology Department, Head and Neck Surgery, Oregon Health & Science University, Portland, OR, United States; ^3^ Department of Cell, Developmental & Cancer Biology, Oregon Health & Science University, Portland, OR, United States

**Keywords:** cancer, intercellular communication, B cells, extracellular vesicles, *in vivo*

## Abstract

Three main areas of research revolve around extracellular vesicles (EVs): their use as early detection diagnostics for cancer prevention, engineering of EVs or other enveloped viral-like particles for therapeutic purposes and to understand how EVs impact biological processes. When investigating the biology of EVs, it is important to consider strategies able to track and alter EVs directly *in vivo*, as they are released by donor cells. This can be achieved by suitable engineering of EV donor cells, either before implantation or directly *in vivo.* Here, we make a case for the study of native EVs, that is, EVs released by cells living within a tissue. Novel genetic approaches to detect intercellular communications mediated by native EVs and profile recipient cells are discussed. The use of Rab35 dominant negative mutant is proposed for functional *in vivo* studies on the roles of native EVs. Ultimately, investigations on native EVs will tremendously advance our understanding of EV biology and open novel opportunities for therapy and prevention.

## Introduction

A key mode of communication between cells involves extracellular vesicles (EVs), which incorporate donor cell-derived signals (both membrane-bound and intracellular) that are delivered to acceptor/recipient cells (1). This process profoundly affects key biological activities, including transfer of processed antigen from activated B cells to follicular dendritic cells in the lymph nodes (2), glucose and lipid metabolism via gut-liver communication (3), synaptic activity and plasticity between neurons and glia (4, 5) and at the feto-maternal interface (6). Consequently, alteration or amplification of EV-mediated intercellular communications foster pathophysiological processes (7). Donor and recipient cells may reside in the same microenvironment, in which case EVs regulate paracrine cell-to-cell communication. EVs may also be distributed systemically, via lymph and blood vessels, and operate as endocrine signals between organs or distant cells (8). Although current approaches involving the isolation and injection of exogenous EVs (that is, from cell cultures or biofluids) in animal models permits fine control of pharmacokinetic and pharmacodynamic parameters, it is not clear whether the information obtained from exogenously administered EVs is adequate to address many aspects of EV biology (9). Thanks to their small size and membrane envelope, EVs can deliver complex and biologically meaningful messages by clustering ligands on their surface and by displaying different signals at once. If EV membranes fuse with recipient cells, EV cargo such as mRNAs and miRNAs is released in the cytoplasm and can extend their biological functions into the recipient cell. However, our knowledge of the cellular and molecular mechanisms that govern cell-cell communication via EVs remains far from comprehensive, at least partly due to technical challenges in tracking and manipulating EVs *in vivo*.

**Figure 1:**
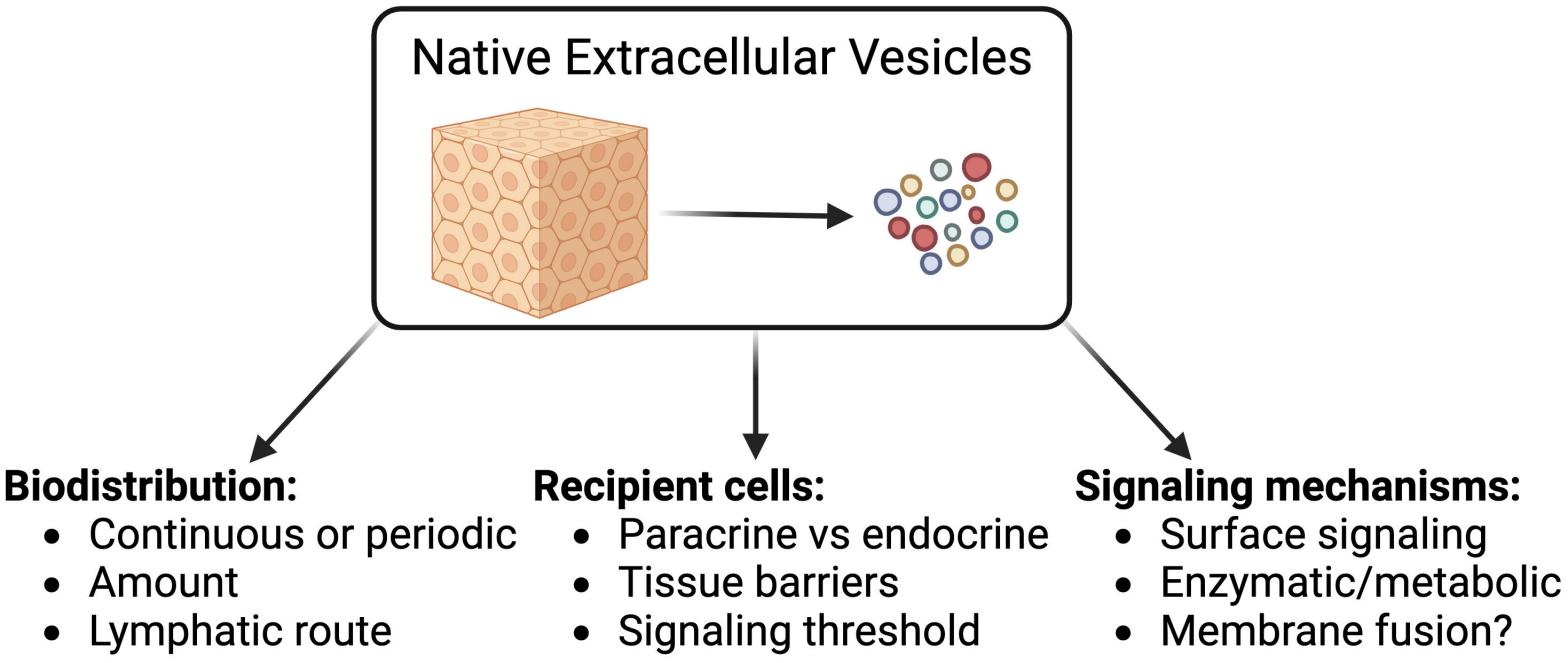
Key knowledge gaps in understanding EV biology that can be tackled by studying native EVs.

In order to advance the field of EV biology, it is crucial to move beyond exogenous administration of EVs, which incompletely mimics physiological EV release and signaling. Physiological and pathological factors that influence EV composition and function, such as nutrients and 3D cellular architectures, are absent or difficult to recapitulate *in vitro* (10, 11). Moreover, *ex vivo* models in which purified EVs are reinfused intravenously, would allow EV subtypes, some of which would normally act locally, to artificially reach non-physiological sites. For example, EVs involved in ECM deposition and modulation might normally act near the cell of origin (12), as would EVs released at immunological synapses (2, 13). In addition, anatomical differences in vascular permeability (for example, liver versus brain), pathological conditions affecting endothelial barrier function (inflammation and cancer), or defense mechanisms restricting EV diffusion within the draining lymph nodes could alter the biodistribution and cellular targets of EVs (14, 15). Thus, a full understanding of EV signaling will require the study of native, endogenous EVs, defined as EVs released by cells living within a tissue (**Figure 1**).

The major knowledge gaps in our understanding of native EV (nEV) contributions to intercellular communication can be classified based on their scale: i) at the organ level, the impact of tissue structures and compartmentalization on the biodistribution of EVs; ii) at the cellular level, the significance of the signals delivered to EV recipient cells; and iii) on a molecular level, the mechanistic details of EV-mediated signal transduction.

### Biodistribution of EVs is affected by biases from EV isolation

The relative contribution of local vs systemic EV-mediated cell-cell communication is largely unknown. In the last decade, studies aiming at defining where EVs diffuse and accumulate in animal models have employed different EV isolation methods (16). Nonetheless, EV isolation *per se* introduces biases (8, 17) and different EV isolation methods may yield different EV subsets (18). Several investigations reported the impact of EVs in co-culture with different recipient cell types, whether they have or not the ability to come into contact with EVs *in vivo* in the first place. Most *ex vivo* studies have reinfused purified EVs via blood circulation, which brings three separate issues: i) a bolus injection of EVs does not recapitulate continuous or periodic release; ii) the amount of EVs injected is arbitrary and in most studies well above physiological levels; and iii) it is assumed that intravenous reinfusion is the proper biodistribution route, while we and others have demonstrated that nEVs first drain into the lymphatics, and, only after passing the filter of lymph node chains, do nEVs join the systemic circulation (15, 19). For these reasons, in order to understand the *in vivo* biology of EVs, it is crucial to develop approaches to track nEVs under physiological conditions.

### Impact of native EVs on recipient cells is unclear

In order to understand the signals that nEVs deliver to target cells, either locally or systemically, it is crucial to determine who are these cellular targets and what impact do nEVs have on them. Given the limited knowledge on EV biodistribution (see point 1), it is unsurprising that the identity of recipient cells targeted by nEVs is also largely unknown. As a consequence, our understanding of the importance of EV-mediated cell signaling is still very rudimentary and mostly derived from artificial model systems. These issues are compounded by the fact that EVs are extremely small [most EV subsets are sub-micron size in diameter (1)], often below the diffraction limit of conventional microscopy (9), and thus, they can carry limited amounts of fluorescent reporters. As a consequence, only recipient cells that bind nEVs in high numbers can be detected and isolated for profiling studies (15, 20). Remote EV-cell communications are much harder to identify as the amount of nEVs exponentially decreases with distance from EV donor cells (21). Therefore, defining the impact of nEVs on the full repertoire of local and distant recipient cells is still an unmet challenge that requires the application of paradigm-shifting technologies (22).

### The mechanisms for EV signal transduction must be validated *in vivo*

Three main mechanisms have been proposed to explain how EVs impact recipient cells. A lot of excitement came from reports describing “horizontal transfer” of bioactive material (including DNA, mRNA and miRNA) between co-cultures of EVs and recipient cells (23). However, evidence of horizontal transfer (or fusion) as a general mode for EV operation *in vivo* is rather scarce, as we and others have reported (15, 17, 24). This is likely due to the fact that endosomal escape is either a rare process or a highly regulated one (25, 26). As a second mechanism, EVs may incorporate active enzymes, which would deliver their enzymatic activity to distant locations (27). A third option is based on classical ligand-receptor interactions between surface proteins and lipids on EVs and transmembrane receptors on recipient cells. In this scenario, EVs represents a key enhancing factor for signaling because they not only allow for clustering of many ligand molecules [which boosts signaling capacity (28)], but they also enable the co-delivery of multiple different signals packaged in the same EV, creating a *de facto* mobile signaling synapse. Lipophilic signaling molecules could similarly be transported via nEV lipid bilayer to alert remote cells (29). It’s important to highlight that surface signaling includes mechanisms where components from the extracellular environment bind to nEVs after being released, forming a so-called EV corona (30). The relative contribution of horizontal transfer, enzymatic activity and signaling synapse is largely unclear.

In this context, EVs may represent emerging targets for the prevention and treatment of diseases that stem from environmental exposures (31). This is because EVs are involved in the clearance and transport of proteins and nucleic acids, responding to cellular stress and unwanted molecules (5, 32, 33). Therefore, a better understanding of nEVs is key to improve disease detection and prevention.

## Recent progress

### Mapping the biodistribution of nEVs

The development of genetic approaches to label and track nEVs promises to revolutionize the field studying EV biology in living organisms (9). We reported for the first time that implanting genetically engineered EV donor cells with bioluminescent EV reporters enables investigations into whole-body biodistribution of nEVs in mice (15). Results from experiments employing this strategy challenged the assumption that tumor-derived EVs directly enter the blood circulation, and instead indicated that lymphatic drainage of nEVs plays an important role in their dissemination (15, 34). These results are consistent with the well-established directionality of interstitial fluid and lymph flow, based on pressure gradients and lymphatic endothelial cell features (35, 36), and suggest that only cell types with access to the systemic circulation (such as endothelial cells) may directly release nEVs into the blood or possibly translocate tissue EVs. The discovery of a lymph-borne biodistribution route for nEVs is foundational for investigations of long-range communication via nEVs, not only in cancer but also during homeostasis and in other conditions, because it maps the barriers encountered by nEVs and short-lists the number of potential recipient cells therein.

### Enabling functional studies of nEVs *in vivo*

In order to perform functional studies, a tool to inhibit the release of nEVs is necessary. Such a tool should be specific enough to selectively block nEVs while sparing other soluble factors secreted by EV donor cells. We and others have previously validated expression of Rab35 dominant-negative (DN) mutant (S22N) as a tool to profoundly reduce (>90%) EV release in multiple cell types (15, 37–39). We recently confirmed that expression of Rab35-DN specifically inhibits nEV release, with minimal impact on other secreted factors like cytokines (37). This is important because it enables us to attribute the biological effects of Rab35^S22N^ expression to lack of nEV signaling. The use of a DN mutant allows to avoid the burden of validation that is required when using RNA interference approaches (40, 41). In *in vivo* models of cellular senescence, inhibition of nEV release via expression of Rab35^S22N^ impacted recruitment of specific immune cell types, namely those expressing major histocompatibility class-II surface receptors (37). Coupling this approach with well-established technologies, such as mouse transgenesis or lentiviral vector-based *in vivo* gene delivery (42), promises to open up new frontiers for exploration of nEVs impacts in homeostasis and disease.

### Hitting the limits of state-of-the-art approaches

By tracking nEV biodistribution, we discovered that draining lymph nodes were a primary site of nEV accumulation (15). When we employed fluorescent reporters to identify nEV recipient cells at these remote locations, we identified a specialized tissue macrophage as the main recipient cell type (15). These macrophages, located in the sub-capsular sinus of lymphoid organs, were known to capture particulate antigens such as viruses, viral-like particles, bacteria and immune-complexes for initiation of humoral immune responses (eg. antibody production) (43–46). These studies suggest that lymph node B cells may be involved in responding to nEVs signaling. If confirmed, the long-range cross-talk between nEVs and B cells would be the first of its kind to be reported. Understanding the significance of nEV-B cell communication is important to elucidate the influence of humoral immunity during homeostasis and disease (47, 48). Our data support this model, since we detected a significant (albeit modest) increase in nEV binding to lymph node B cells upon depletion of sub-capsular sinus macrophages, and inhibition of nEV release partially reverted the impact of B cells on disease progression (15). However, the signal intensity provided by current genetic EV reporters was not enough to isolate lymph node B cells interacting with nEVs.

### Development of next-generation nEV reporters

To increase detection sensitivity for nEV recipient cells, we reasoned that, instead of tagging EVs themselves (first-degree labeling), an approach able to tag recipient cells via nEV binding (second-degree labeling, or “EV painting”) would allow us to take advantage of the much larger surface of the recipient cell for reporter accumulation and ultimately would enable isolation and profiling of nEV recipient cells. To this end, we adapted an interaction-based reporter system composed of a transpeptidase enzyme (SortaseA) and its consensus peptide substrate (LPETGS) (22). SortaseA catalyzes the formation of a peptide bond between the consensus peptide and a nearby protein containing an N-terminal glycine residue (49). More than 100 endogenous cell surface proteins contain N-terminal glycine residues in mice, including ubiquitously expressed proteins like histocompatibility antigen receptors and adhesion molecules (50). We selected a SortaseA-based system because it has several advantages over other approaches for studying cell-cell interactions: i) SortaseA labeling is not binary and does not require computational deconvolution, in contrast to PIC-seq (51); ii) SortaseA affinity for its consensus peptide is in the millimolar range and requires *de facto* binding (that is, proximity less than 15 nm between nEV and recipient cell membranes (52), thereby enhancing labeling specificity compared to synNotch system which has nanomolar affinity (53). We designed a membrane-bound form of SortaseA that is seamlessly packaged into EVs, independent of EV donor cell type and without evident alterations in EV biogenesis (22). Upon comparison with a reference EV reporter (CD63-GFP fusion), SortaseA+ EVs generated a signal intensity more than 10-fold higher on EV recipient cells (22). The SortaseA-based nEV reporter allowed us to study cancer stem cell-derived nEVs *in vivo*, within the stem cell niche (54). Future studies using transgenic mice expressing the EV-targeted SortaseA will enable single cell profiling of nEV-recipient cells.

**Figure 2:**
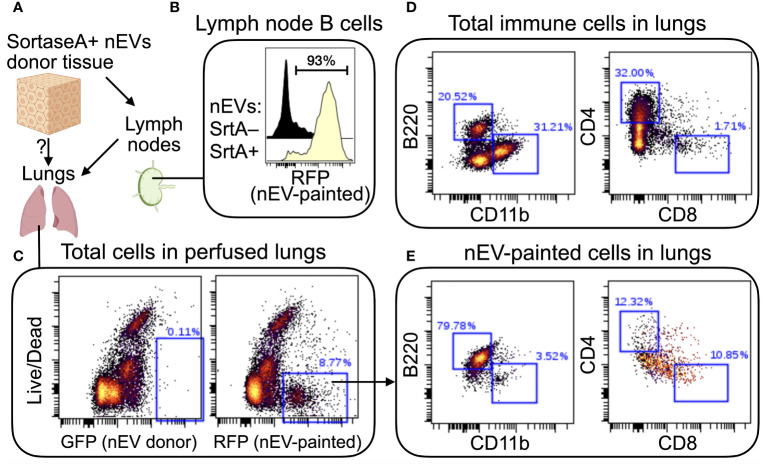
*In vivo* demonstration of long-range cell-cell signaling via nEVs. **(A)** Schematic of the approach. Syngeneic squamous carcinoma cells (MOC2) were engineered to express a membrane-bound form of SortaseA (for inclusion in EV membranes) and to secrete a red fluorescent protein (RFP: mScarlett) tagged with the aminoacid sequence LPETGG (the SortaseA recognition signal). Engineered MOC2 were implanted in the skin of immunocompetent mice (C57B/6). Draining lymph nodes and distant organs, including lungs, were analyzed 21 days later. **(B)** SortaseA+ nEVs accumulated in draining lymph nodes, as expected, where they mainly engaged in cross talks with local B cells. Since tumor cells also express the SortaseA substrate (which is secreted into the extracellular environment and drains into the lymph nodes), when local B cells interact with SortaseA+ nEVs, the nEV-bound enzyme has continuous access to its substrate. Control mice received SortaseA-negative cells. **(C)** Confirmation that nEV-donor cells did not migrate to the lungs (left plot) and that “EV-painted” cells (that is, cells that have experienced nEV binding) were present in perfused lungs. **(D, E)** Comparison of lung-resident total immune cells **(D)** with nEV-painted cells **(E)** highlights a strong enrichment for B cells (B220+CD11b-CD4-CD8-) among the latter. N=2.

To demonstrate feasibility of using the SortaseA-based nEV reporter *in vivo*, we aimed to demonstrate presence of EV-painted cells in distant organs of mice receiving a skin implant of SortaseA+ nEV donor cells (**Figure 2**). As expected, nEVs collected in draining lymph nodes, where we detected a strong signal on all B cells, indicating that, at some point, they bound nEVs (**Figure 2B**). Strikingly, when we analyzed other non-immune organs after perfusion (to remove circulating cells, **Figure 2C**), we found a significant fraction of lung-resident B cells displaying the mark of interactions with skin nEVs (compare **Figures 2D, E**). Pulse-chase experiments will address whether these nEV-experienced B cells in the lungs have migrated there from lymph nodes or if they were labeled by circulating nEVs. These data indicate that the SortaseA-based reporter is a viable approach for sensitive detection of long-range cross talk via nEVs. Overall, these results support the idea that nEVs are a type of particulate antigen that signals to B cells located in remote lymphoid organs.

## Conclusions

There is a case to be made for the study of native EVs, defined as EVs released by cells living within a tissue. The EV community is embracing the importance of studying nEVs to advance our understanding of their biology (55). New biological insights from investigation of nEV in diagnosis of neurodegenerative diseases (56) and cancer (57, 58) are poised to improve prevention strategies. Although innovative approaches that take advantage of nEVs are emerging (59), more studies are needed to unravel the breath of nEV impact in disease. The technologies described in this perspective will support these efforts. Ultimately, coupling advanced EV engineering with mouse transgenesis and modern sequencing technologies will tremendously benefit our understanding of EV biology.

## Data availability statement

The raw data supporting the conclusions of this article will be made available by the authors, without undue reservation.

## Ethics statement

The animal study was approved by Stanford Cancer Institute. The study was conducted in accordance with the local legislation and institutional requirements.

## Author contributions

DN: Investigation, Writing – review & editing. QL: Funding acquisition, Writing – review & editing. FP: Conceptualization, Data curation, Formal analysis, Funding acquisition, Investigation, Methodology, Project administration, Resources, Supervision, Validation, Visualization, Writing – original draft, Writing – review & editing.
